# Experience with a single dose of recombinant activated factor VII for the management of mild-to-moderate bleeds in haemophilia

**DOI:** 10.1111/j.1365-2516.2010.02403.x

**Published:** 2011-03

**Authors:** D ALMAGRO, O AGRAMONTE, D CASTILLO, Y ZAMORA, J M BALLESTER

**Affiliations:** Hemostasis Department of Hematology and Immunology, Institute of Hematology and ImmunologyLa Habana, Cuba

The development of inhibitors to factor VIII or factor IX remains one of the most challenging complications in the management of haemophilia, and greatly compromises the efficacy of replacement therapy. Despite the option of inhibitor eradication via immune tolerance therapy, it is estimated that around 30% of patients do not respond to immune tolerance therapy and will have inhibitors for life [1].

Today, recombinant activated factor VII (rFVIIa; NovoSeven®, Novo Nordisk, Bagsværd, Denmark) is considered the first-line treatment option for controlling bleeding in haemophilia patients with inhibitors [2]. Standard dosing of rFVIIa facilitates haemostasis by activating factor X directly on the platelet surface, thereby bypassing the tenase complex. At higher rFVIIa doses the action is increased further, resulting in a more stable haemostatic plug with greater resistance to fibrinolysis [3]. There has, therefore, been debate over the most effective dosing regimen for rFVIIa in the management of bleeding episodes in haemophilia patients with inhibitors, and in particular, interest in the use of a single, higher dose treatment that may offer the same haemostatic benefit as the traditional standard dosing regimen of rFVIIa 90 μg kg^−1^ given every 2–3 h, but in a more convenient dosing regimen.

Here, we report our experience on the use of an initial single bolus dose of rFVIIa (180 μg kg^−1^ in children and 150 μg kg^−1^ in adults) for the management of mild-to-moderate bleeding episodes in haemophilia A patients with inhibitors.

To be eligible for inclusion in the study, patients had to have a confirmed diagnosis and history of haemophilia with inhibitors and mild-to-moderate bleeds. Patients gave informed consent (for children the parent or legal guardian provided this) to receive treatment with rFVIIa. All enrolled patients were treated within 12 h of the onset of a bleeding episode. Patients were excluded from the study if they had severe bleeds, presented for treatment more than 12 h after the onset of a bleeding episode, or could not be shown to have haemophilia with inhibitors.

Between March 2006 and January 2008, a total of 35 bleeding episodes in haemophilia A patients with inhibitors were treated with rFVIIa, 27 episodes in five children up to 14 years of age (180 μg kg^−1^) and eight episodes in three adult patients (150 μg kg^−1^). The use of a higher dose of rFVIIa in children reflects the known faster clearance of rFVIIa in paediatric patients.

Bleeding episodes were moderate in 21 (60%) cases and mild in 14 (40%) cases. All doses of rFVIIa were given as a single bolus injection. If bleeding persisted after initial dosing at either 180 μg kg^−1^ or 150 μg kg^−1^, a further bolus dose of rFVIIa (90 μg kg^−1^) could be given at 3-hourly intervals until haemostasis was achieved.

Treatment response to rFVIIa dose was classified as good, partial or lack of response. A good response was bleeding that stopped following the initial single dose of 180 or 150 μg kg^−1^ rFVIIa; a partial response was bleeding that stopped following a second dose of 90 μg kg^−1^ rFVIIa; and a lack of response was characterized when bleeding symptoms persisted after administration of two doses of rFVIIa. Treatment was considered effective if bleeding resolved with up to two doses of rFVIIa (good and partial responses). Treatment was considered ineffective if more than two doses of rFVIIa were needed to stop the bleeding (lack of response to rFVIIa doses). Additional assessments made during the study included clinical evaluation of pain, pain relief and tenderness as reported by the patients, plus measurable changes in haematoma size, joint mobility and bleeding arrest. These parameters were evaluated at presentation and 3 h after each rFVIIa infusion.

A single dose of rFVIIa 180 μg kg^−1^ stopped mild-to-moderate bleeding in 59% of bleeding episodes in children. Haemostasis was achieved after a second dose of 90 μg kg^−1^ rFVIIa in a further 37% of bleeding episodes. One bleeding episode in a child did not stop after two doses of rFVIIa (4%). In adult patients, a good response was achieved in 37.5% of cases. Haemostasis was achieved after a second dose of 90 μg kg^−1^ rFVIIa in the remaining 62.5% of bleeding episodes (Table 1). The response rate was lower in adults, probably because a higher dose of rFVIIa is necessary for a further increase of thrombin generation. In addition, it is likely that the adults had worse arthropathy than the children, which may be another reason for their lower response. The bleeding response was rated as good in 71% of patients with mild bleeds and in 42% of those with moderate bleeds. Overall, in 97% of bleeding episodes the event stopped with only two doses of rFVIIa (average 1.5 doses). In the 29 joint bleeds, pain and tenderness disappeared and joint mobility improved after the first dose of rFVIIa in 18 cases (62%). The remaining haemarthroses responded to a second dose. In all three reported haematomas, the pain disappeared and haematoma size diminished after two doses of rFVIIa.

**Table 1:** Treatment response to rFVIIa doses in inhibitor patients with mild-to-moderate bleeds

		Response to rFVIIa, *n* (%)		
		
	rFVIIa dose (μg kg^−1)^	Good	Partial	Lack of response
Children (*n*=**27)	180	16 (59)	10 (37)	1 (4)
Adults (*n*=**8)	150	3 (37.5)	5 (62.5)	
Total (*n*=**35)		19 (54)	15 (43)	1 (3)

*n*, number of bleeding episodes.

Good, haemostasis achieved with initial single dose of rFVIIa; Partial, haemostasis required an additional 90 μg kg^−1^ dose of rFVIIa; Lack of response, bleeding continued after two doses of rFVIIa.

It was noted that the response to rFVIIa varied in the three patients with oral bleeding. In one case, arrest of bleeding was achieved with the first dose of rFVIIa, whereas a second dose of rFVIIa was required for the second case, and the remaining patient started to re-bleed within 24 h, despite attaining full haemostasis initially.

No adverse events were reported during the administration of rFVIIa in this patient cohort.

The recent literature contains a number of reports of the use of a higher and single dose of rFVIIa to achieve bleeding control in haemophilia patients with inhibitors. Overall, these studies demonstrate that a single dose of rFVIIa 270 μg kg^−1^ optimizes on-demand treatment by offering the same level of efficacy and safety as dosing schedules of rFVIIa 90 μg kg^−1^ given every 2–3 h [4,5].

The results of this study support the view that a single high dose of rFVIIa offers a viable and effective alternative to standard dosing without altering the safety profile. Although this was a small study which used single doses of rFVIIa that are lower than those recently shown to be both effective and well tolerated by adults and children with haemophilia with inhibitors, it highlights the importance of optimizing the dose regimen according to local resources and patient characteristics, such as age, with the possibility of reducing the cost of the treatment.
